# Environmental Stress-Dependent Effects of Deletions Encompassing *Hsp70Ba* on Canalization and Quantitative Trait Asymmetry in *Drosophila melanogaster*

**DOI:** 10.1371/journal.pone.0017295

**Published:** 2011-04-25

**Authors:** Kazuo H. Takahashi, Phillip J. Daborn, Ary A. Hoffmann, Toshiyuki Takano-Shimizu

**Affiliations:** 1 Department of Population Genetics, National Institute of Genetics, Mishima, Shizuoka-ken, Japan; 2 Centre for Environmental Stress and Adaptation Research and Department of Genetics, The University of Melbourne, Parkville, Melbourne, Australia; 3 Department of Genetics, Graduate University for Advanced Studies (SOKENDAI), Mishima, Shizuoka, Japan; 4 Department of Biosystems Science, Graduate University for Advanced Studies (SOKENDAI), Hayama, Kanagawa, Japan; 5 Department of Biological Science, Graduate School of Science, The University of Tokyo, Tokyo, Japan; Harvard University, United States of America

## Abstract

*Hsp70* genes may influence the expression of wing abnormalities in *Drosophila melanogaster* but their effects on variability in quantitative characters and developmental instability are unclear. In this study, we focused on one of the six *Hsp70* genes, *Hsp70Ba*, and investigated its effects on within-and among-individual variability in orbital bristle number, sternopleural bristle number, wing size and wing shape under different environmental conditions. To do this, we studied a newly constructed deletion, *Df(3R)ED5579*, which encompasses *Hsp70Ba* and nine non-*Hsp* genes, in the heterozygous condition and another, *Hsp70Ba^304^*, which deletes only *Hsp70Ba*, in the homozygous condition. We found no significant effect of both deletions on within-individual variation quantified by fluctuating asymmetry (FA) of morphological traits. On the other hand, the *Hsp70Ba^304^/Hsp70Ba^304^* genotype significantly increased among-individual variation quantified by coefficient of variation (CV) of bristle number and wing size in female, while the *Df(3R)ED5579* heterozygote showed no significant effect. The expression level of *Hsp70Ba* in the deletion heterozygote was 6 to 20 times higher than in control homozygotes, suggesting that the overexpression of *Hsp70Ba* did not influence developmental stability or canalization significantly. These findings suggest that the absence of expression of *Hsp70Ba* increases CV of some morphological traits and that HSP70Ba may buffer against environmental perturbations on some quantitative traits.

## Introduction

Based on the observation that traits often lacked variability, Waddington [1] proposed the notion of “canalization” as an ability of a genotype to produce relatively constant phenotypes across different environments and genetic backgrounds. The evolution of mechanisms that reduce phenotypic variability under environmental and genetic perturbations can be facilitated by stabilizing or fluctuating selection [2], [3], because genotypes that produce a fit phenotype with high reproducibility would be superior to ones with low reproducibility [4], [5]. Canalization is often quantified as variation of a phenotype among individuals that share genetic or environmental backgrounds [6], [7], [8], using measures for group-level variation of traits of interest.

Closely tied to the notion of canalization is the idea of developmental instability, the random deviations from canalized phenotypes within an individual as opposed to variation among individuals. Developmental instability is commonly measured by fluctuating asymmetry (FA), random deviations from perfect bilateral symmetry [9], [10], [11], [12], [13]. FA of various morphological characters, such as bristle traits and wing morphology, has been studied to examine the effect of genetic and environmental perturbations on developmental stability in *D. melanogaster* [7], [9], [10], [11]. Debat *et al.* [14] showed that FA in wing shape may be a particularly sensitive indicator of stressful environmental perturbations. Wing shape can vary in more flexible ways and be more informative than simple traits such as size of body parts or number of bristles [10].

The molecular mechanism of canalization and developmental instability remained unknown until Rutherford and Lindquist [15] showed that both the genetic and pharmacological inactivation of HSP90 exposes hidden phenotypic variation in *Drosophila melanogaster*. Ensuing studies using *Arabidopsis thaliana* supported the idea that *Hsp90* is involved in canalization [16], [17], [18]. HSP90 is a molecular chaperone, and most of its identified cellular targets are involved in signal transduction and chromatin organization [19], [20], [21], [22]. Given its function as a molecular chaperone, *Hsp90* may contribute to the stability of those processes under environmental and genetic perturbations. Temperature sensitivity of the phenotypic variability in *Hsp90* mutants suggests that normally functioning *Hsp90* buffers a thermal perturbation on developmental processes [15], [18], [23]. However, the inhibition of HSP90 does not always influence canalization or developmental instability of traits, particularly quantitative traits in *Drosophila* [9], [10], [24].

Several other molecular chaperones respond to environmental stresses in the *D. melanogaster* genome, and among them, *Hsp70* is one of the well-studied genes. HSP70 is inducible by thermal and nutritional stresses and even inbreeding, and is involved in stress resistance [25], [26], [27], [28], [29]. Roberts and Feder [30] examined the effect of the copy number of *Hsp70* genes on developmental processes and observed a reduction in developmental abnormalities in a transgenic strain with an increased copy number of *Hsp70*. On the other hand, Williams et al. [31] found that strains with increased HSP70 levels had an increased incidence of developmental wing abnormalities. These studies suggest that *Hsp70* genes may influence canalization.

In this study, we focus on the potential effects of an *Hsp70* gene on canalization and developmental instability (measured as FA) for wing shape and other traits. There are six nearly identical genes that encode HSP70 in *D. melanogaster*, and they are located at cytological loci 87A and 87C on the right arm of chromosome *3* [32]. Previous studies did not distinguish six different *Hsp70* genes and examined their effect collectively [30], [31]. However, they may have different functions even if sequence homology is quite high among them. *Hsp70Ba*, one of the *Hsp70* genes, is known to be polymorphic in natural *D. melanogaster* populations and considered a target of selection for thermotolerance and resistance to other stresses [33], suggesting that its function as a molecular chaperone is important for survival. If *Hsp70Ba* is involved in canalization or developmental stability, loss of the *Hsp70Ba* gene or change in its expression level may increase CV and FA of morphological traits. We have therefore targeted specifically *Hsp70Ba* in this study. To this end, we used two independent *Hsp70Ba* deletions, a newly constructed deletion encompassing *Hsp70Ba* and several other genes, and the one constructed by Gong and Golic [32], and examined their effects on developmental processes and stability under different environmental stresses. We tested their effects on mean values, CV and FA for variable traits such as bristle number, wing size and shape under thermal and nutritional stresses by comparing the deletion heterozygotes or homozygotes and control homozygotes with an isogenic background.

## Methods

We used the DrosDel isogenic deficiency kit and RS element-FLP system to generate a deletion [34]. This approach allows the end points of the deletions to be determined with single-base-pair resolution. Ryder *et al.* [34] constructed a control strain (DSK001) that was isogenic for the *X*, second and third chromosomes, and used it to create RS element inserted strains. The control and all the deficiency lines have an isogenic background except for deletions. We generated a 33.6 kb deletion encompassing *Hsp70Ba* using FRT recombination between 5-SZ-3266 and CB-0592-3 RS elements [34], [35]. The resulting *Df(3R)ED5579*, the smallest possible deletion encompassing *Hsp70Ba* with the deficiency kit, was confirmed by the polymerase chain reaction (PCR) method. The deletion, *Df(3R)ED5579*, includes nine non-*Hsp* genes. Molecular functions of seven of them are still unknown. One of the two known genes, *desat1*, is involved in cuticular hydrocarbon synthesis [36], [37]. The other one, CG12267, is suggested to have RNA polymerase activity, but its phenotypic effect when it is deleted is unknown. Based on this information and the known effects of *Hsp70*s on morphological trait variation, *Hsp70Ba* is the most likely candidate gene for the bristle number and wing morphology traits considered here, although this would require further validation. Because the deletion was homozygous lethal, we maintained the deletion strain with a balancer, *TM6C*. For trait comparisons, we used heterozygotes of the *Df(3R)ED5579* and DSK001 chromosomes (*Df(3R)ED5579*/DSK001), as well as homozygotes of DSK001 chromosomes (DSK001/DSK001). Because maternally produced HSP70Ba might influence developmental pathways in offspring at an early stage, we crossed the deletion (*Df(3R)ED5579*) and control (DSK001) strains reciprocally, and used two reciprocal deletion heterozygotes for experiments.

Gong and Golic [32] constructed an *Hsp70Ba* deletion strain (*w^1118^*; +; *Hsp70Ba^304^* from the Bloomington Drosophila Stock Center) using ends-out targeting. The deletion encompasses only *Hsp70Ba*, but there is no isogenic control strain to compare the effect of the deletion because, in the process of gene targeting, many crosses with different strains were performed. We used a double balancer strain (*w^1118^*; *If*/*CyO*; *MKRS*/*TM6B*) to replace the 2 nd chromosome of the *Hsp70Ba* deletion strain (*w^1118^*; +; *Hsp70Ba^304^*) with the one of the control strain (*w^1118^*; DSK001; DSK001) to obtain the experimental genotype *w^1118^*; DSK001; *Hsp70Ba^304^*. *Hsp70Ba^304^* was homozygous viable, so we used homozygotes (*Hsp70Ba^304^/Hsp70Ba^304^*) in further experiments.

### Constant condition experiment

We investigated the combined effect of different stresses and deletions on morphological traits under several nutritional and thermal conditions throughout their life. We used two fly media with different nutritional conditions. Standard medium was made of water (1000 ml), dried yeast (35 g), soy flour (20 g), cornmeal (73 g), agar (24 g), malt extract (46.25 g), and dextrose (75 g) for a one liter cook, and the mixture was boiled well. Then we added the 13.75 ml of the total standard medium volume of acid mix (412 ml propionic acid plus 42 ml orthophosphoric acid made up to 1 litre in water) and 16.5 ml of nipagin (100 g methyl-p-hydroxybenzoate 1 litre in 90% ethanol). The standard medium was used for ‘rich’ condition, and medium with half the amount of dextrose and one tenth the amount of yeast compared to the rich medium was used for the ‘poor’ condition. In the case of *Df(3R)ED5579*, a hundred eggs were collected from crosses between the deletion (*w^1118^*; DSK001; *Df(3R)ED5579*/*TM6C*) and control (*w^1118^*; DSK001; DSK001) strains to obtain *w^1118^*; DSK001; *Df(3R)ED5579*/DSK001. In the case of *w^1118^*; DSK001; *Hsp70Ba^304^* and the control strain *w^1118^*; DSK001; DSK001, a hundred eggs were collected from each strain separately. Eggs were introduced into each vial with rich or poor medium and reared under different thermal conditions (18°C, 23°C or 28°C) in incubators until they were collected for morphological measurement. Three to five replication vials were set up for each condition, and three females and males from each vial were sampled for the morphological measurement. The time and number of emerging adults were recorded every day, and then flies were preserved in 70% ethanol for morphological measurements. For *Hsp70Ba^304^/Hsp70Ba^304^*, because viability was very low at 18 and 28°C and under nutritional stress, only flies emerging at 23°C from the rich nutritional medium were measured. Because sampling three females and males from each vial is not enough to evaluate CV of morphological traits accurately, we sampled five to seven extra individuals from each vial for measurement of the morphological traits on the right side of the body.

### Short-term heat stress experiment

Buffering at a specific time during development might be important for developmental stability, so we evaluated the effect of a short-term exposure to a heat stress on morphological traits at the five times: one, three, five, seven, or nine days after the egg introduction (DAEI). This approximately corresponded to developmental stages as follows: first instar larval stage at 1DAEI, second instar larval stage at 3DAEI, third instar larval stage at 5DAEI, late third instar larval stage to prepupal stages at 7DAEI, and pupal stage at 9DAEI. For each time period, a hundred eggs were collected as above and introduced into each vial for exposure to a heat stress at 37°C in a water bath for one hour. We used only rich nutrition medium to exclude nutritional stress. Three to five replication vials were set up for each condition, and three females and males from each vial were sampled for morphological measurement. As above, date and the number of adults emerged were recorded, and flies were preserved in 70% ethanol. For accurate CV estimation, five to seven extra individuals were sampled for morphological measurement.

### Quantitative RT-PCR

To examine the relative expression level of *Hsp70Ba* in the *Df(3R)ED5579/*DSK001 and control strain (DSK001/DSK001), and also the effect of the deletion on the expression of other *Hsp* genes such as *Hsp22*, *Hsp70* other than *Hsp70Ba* (*Hsp70*s), and *Hsp83*, we conducted quantitative RT-PCR. We crossed deletion heterozygotes, *w^1118^; DSK001; Df(3R)ED5579/TM6C*, and *w^1118^*; DSK001; DSK001/*TM6B* to replace *TM6C* with *TM6B* and obtained *w^1118^; DSK001; Df(3R)ED5579/TM6B*, and then, crossed it to the control *w^1118^*; DSK001; DSK001 to obtain the experimental genotype *w^1118^*; DSK001; *Df(3R)ED5579*/DSK001 larvae. To expose control larvae to the same level of competition, we crossed *w^1118^*; DSK001; DSK001 to *w^1118^*; DSK001; DSK001/*TM6B* to obtain experimental *w^1118^*; DSK001; DSK001. Because *Hsp* genes are known to increase their expression levels in response to environmental stresses, a difference in expression levels between DSK001/DSK001 and *Df(3R)ED5579/*DSK001 is expected to be larger under a stressful condition. To give larvae a nutritionally and thermally stressful condition, we collected 100 eggs from the crosses and reared the larvae under poor nutrition at 28°C. About 20 deletion heterozygotes, *Df(3R)ED5579*/DSK001, and the control homozygotes DSK001/DSK001 were sampled at five and seven DAEI, and three replicate groups were collected. Total RNA was isolated from the larvae using TRIzol reagent (GIBCO/BRL). Each RNA sample was tested for DNA contamination with PCR, and then reverse transcription was performed on 0.5 *µ*g of each RNA sample in a 20 *µ*l reaction using Superscript III Reverse Transcriptase (Invitrogen Life Technologies) and oligo (dT)_20_ primer, following the supplier's instructions. Then, 1 *µ*l of a 1 in 10 dilution of cDNA was used in RT-PCR. RT-PCR was conducted on a RotorGene-3000 (Corbett Research, Sydney) using a QuantiTect SYBR Green PCR kit (Qiagen, Valencia, CA). The PCR conditions and primers for an internal control, a housekeeping gene *RpL11*, were described in Bogwitz *et al.* [38]. We designed PCR primers for all the *Hsp70* genes except for *Hsp70Ba* using a common sequence among them, and also the ones specifically for *Hsp70Ba* using an *Hsp70Ba* specific gap to distinguish its expression from other *Hsp70*s. PCR primers used for *Hsp22*, *Hsp70*s, *Hsp83*, and *Hsp70Ba* were Hsp22F (ATC AAT ATA CCC TTG GAT GAA CT) and Hsp22R (ATT TTC TCT GCT TCC AAG ACT), Hsp70sF (TGA AGA CAA GAA GAG AAC TCT G) and Hsp70sR (CCA GAT CGA TTC CAA TAG CA), Hsp83F (AAA CAT ACA TAC AAG ATG CCA GAA) and Hsp83R (ACT CAT AGC GGA TCT TGT CC), and Hsp70BaF (GAG AAT ACT TTC AAC AAG TTA C) and Hsp70BaR (CAT TGG CGA TAA TCT CAA CCT), respectively. For every experiment, we made standard curves for each gene from a reference sample of cDNA using duplicated serial dilutions with five different cDNA concentrations covering 100-fold concentration range. Standard curves were used to quantify amounts of target and housekeeping transcripts in each sample. We list genotypes and the sources of strains for all the experiments in Table 1.

**Table 1:** List of the genotypes of the flies used for experiments and strains used to construct them.

Experiments	Treatment	Genotype	Strains used to construct experimental flies
Constant condition	Deletion	*w^1118^*; DSK001; *Df(3R)ED5579*/DSK001	*w^1118^*; DSK001; DSK001, *w^1118^*; DSK001; *Df(3R)ED5579*/*TM6C*
		*w^1118^*; DSK001; *Hsp70Ba^304^*	*w^1118^*; DSK001; DSK001, *w^1118^*;+; *Hsp70Ba^304^*, *w^1118^*; *If*/*CyO*; *MKRS*/*TM6B*
	Control	*w^1118^*; DSK001; DSK001	*w^1118^*; DSK001; DSK001
Short-term heat stress	Deletion	*w^1118^*; DSK001; *Df(3R)ED5579*/DSK001	*w^1118^*; DSK001; DSK001, *w^1118^*; DSK001; *Df(3R)ED5579*/*TM6C*
		*w^1118^*; DSK001; *Hsp70Ba^304^*	*w^1118^*; DSK001; DSK001, *w^1118^*;+; *Hsp70Ba^304^*, *w^1118^*; *If*/*CyO*; *MKRS*/*TM6B*
	Control	*w^1118^*; DSK001; DSK001	*w^1118^*; DSK001; DSK001
Quantitative RT-PCR	Deletion	*w^1118^*; DSK001; *Df(3R)ED5579*/DSK001	*w^1118^*; DSK001; *Df(3R)ED5579*/*TM6C*, *w^1118^*; DSK001; DSK001/*TM6B*, *w^1118^*; DSK001; DSK001
	Control	*w^1118^*; DSK001; DSK001	*w^1118^*; DSK001; DSK001

### Measurement of morphological traits

**Figure 1:**
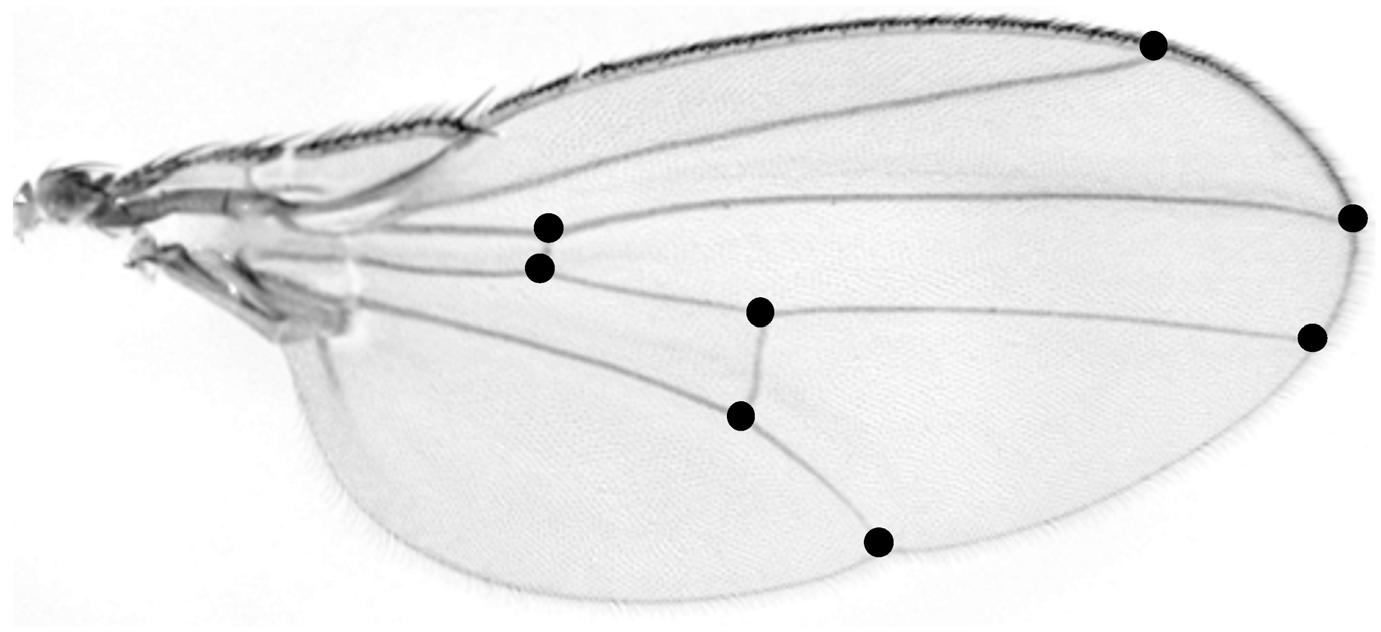
Positions of eight wing landmarks used in this study.

We measured both meristic and metric traits, which may respond differently to a lack of canalization (Kellerman *et al.*, 2007). To evaluate the effect of the deletion on meristic traits, we scored bristle traits, the numbers of orbital bristles (OR) and sternopleurals (SP) on the right and left side of each fly. For metric traits, we measured an allometric component of wing trait, centroid size (CS), and a non-allometric component, wing shape (WS), using the eight landmarks placed on the junctions between longitudinal veins and cross veins or wing margins (Figure 1). We first removed right and left wings, captured their images with an Olympus DP20 CCD camera, and obtained the *x* and *y* coordinates of each landmark with the TPSdig2 program (http://life.bio.sunysb.edu/morph/). We repeated the process of digitizing the landmark coordinates twice for all the samples to evaluate repeatability and used the mean coordinates for the following procedure. For each landmark configuration, the centroid size (CS-the square root of the sum of squared distances from each landmark to centroid) was calculated and scaled to unity. Then, the centroid of each configuration was superimposed onto the centroid of the consensus configuration, and the configurations were rotated so as to minimize the distances between the corresponding landmarks. This Procrustes generalized least squares procedure [39], [40], [41] was conducted with the ‘shapes’ package of statistical software R to obtain Procrustes coordinates. All the analyses were conducted for females and males separately for both constant condition and short-term heat stress experiments.

To evaluate the effect of the deletions on canalization, we calculated CVs of bristle traits and CS for the control and the *Df(3R)ED5579/*DSK001, and *Hsp70Ba^304^/Hsp70Ba^304^* genotypes. We calculated a variance and a mean of each morphological trait for each replicate vial using eight to 10 females or males, and used this to compute CVs for three to six vials. For this calculation, we used the morphological traits on the right side of the body.

For bristle traits and CS, because a size effect was detected in some cases in a preliminary analysis, FA was evaluated as |L-R|/(L+R)/2, where L indicates a trait value on a left side of the body and R on a right side of the body. We also confirmed that there was no significant directional asymmetry for those traits by sign test. For WS, we used a univariate measure of FA devised by Klingenberg and Monteiro [42] (WS1) based on the idea of one-sample standard distance [43], [44], which is equivalent to the one-sample version of the Mahalanobis distance [45]. This measure of FA automatically provides a correction for directional asymmetry [42]. We also defined wing shape FA as the sum of Euclidean distances in Procrustes coordinates between correspondent landmarks of right and left wings [46] (WS2).

### Repeatability and measurement error

To evaluate the measurement error in landmark acquisition, we calculated repeatability (*R*) for each landmark coordinate and CS [47]. With one-way ANOVAs on the repeated measures with individual as a fixed factor, we can attain a ratio *R* from the variance due to differences among individuals to the total variance when the number of repeated measure per individual is two: 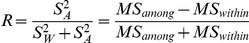 where *S* ^2^_A_ is the among-individual variance component,*S* ^2^_W_ is the within-individuals variance component, and MS is the mean square. This repeatability parameterizes the proportion of variance due to variation between individuals: zero indicates that all variance is attributable to variance within individuals (i. e., 100% measurement error), and one indicates all variance is found between individuals (i. e., 0% measurement error).

To justify further statistical analyses on FA of CS, we evaluated whether between-sides variation is significantly larger than the measurement error with simple two-way ANOVA [13]. We conducted ANOVA for FA of CS with independent variables of side, individual and a two-way interaction term between them for both the constant condition experiment and short-term heat stress experiment.

To assess the relative amounts of directional asymmetry (DA), FA, and measurement error in wing shape variation, we employed Procrustes ANOVA [48] with degrees of freedom under the isotropic model [49]. In this analysis, we included individual, and side and their interaction terms, and added sums of squares across landmarks and coordinates, assuming equal and isotropic variation at each landmark.

### Analysis

To evaluate the effect of nutrition, temperature and the deletions on mean trait values in the constant condition experiment, we performed ANOVAs with number of OR, SP or mean CS averaged for individuals in each vial as a dependent variable, and with nutrition (Rich/Poor), temperature (18°C/23°C/28°C), and deletion (Control/Deletion) as independent variables. Because the *Hsp70Ba^304^/Hsp70Ba^304^* was only recovered at one temperature and nutritional condition, we only had the deletion as a factor in the ANOVA with these lines. For the short-term heat stress experiment, we performed ANOVA with heat shock timing (HST: 1, 3, 5, 7, 9 DAEI) and deletion as independent variables. We applied Bonferroni correction to account for multiple tests performed for each trait for each sex.

We performed thin-plate spline analysis to visualize the effect of treatments and the deletion. Thin-plate splines indicate the differences in two configurations of landmarks as a continuous deformation using regression functions in which corresponding landmarks are matched between configurations to minimize the bending energy [50]. Bending energy is the energy required to bend an infinitely thin metal plate over one set of landmarks so that the height over each landmark is equal to the coordinates of the corresponding landmark in the other configuration [51]. We compared the mean wing shape of each category of each treatment to an overall mean shape by exaggerating their difference by 30-fold, 20-fold, or no exaggeration for graphical display depending on the degree of difference. We performed the thin-plate spline analysis only for the deletion heterozygote.

To evaluate the effect of the treatments and the deletion heterozygote on CV, we performed ANOVA using CV of numbers of OR, SP, or CS as a dependent variable. For the constant stress experiment, nutrition, temperature, deletion and all interaction terms were included as independent variables. For the short-term heat stress experiment, HST, deletion and the interaction terms between them were included as independent variables. For the deletion homozygote, we tested the deletion effect via ANOVAs on FA of OR, SP, CS or WS. We confirmed that all FA traits were normally distributed at vial level by Kolmogorov-Smirnov tests. We applied Bonferroni correction to account for multiple tests performed for each trait for each sex.

To evaluate the effect of the treatments and the deletion heterozygote on FA, we conducted ANOVA using FA of numbers of OR, SP, or FA of CS, WS1 or WS2 as a dependent variable. For the constant stress experiment, nutrition, temperature, deletion and all interaction terms were included as independent variables. For the short-term heat stress experiment, HST, deletion and the interaction terms between them were included as independent variables. For the deletion homozygote, we tested the deletion effect via ANOVAs on FA of OR, SP, CS or WS. All FA traits were normally distributed by Kolmogorov-Smirnov tests. We applied Bonferroni correction to account for multiple tests performed for each trait for each sex.

Sex difference in morphological traits was detected in preliminary analyses, but because the sex difference is not the main focus of this study, we performed all the analyses separately for the sexes.

## Results

### Quantitative RT-PCR

**Figure 2:**
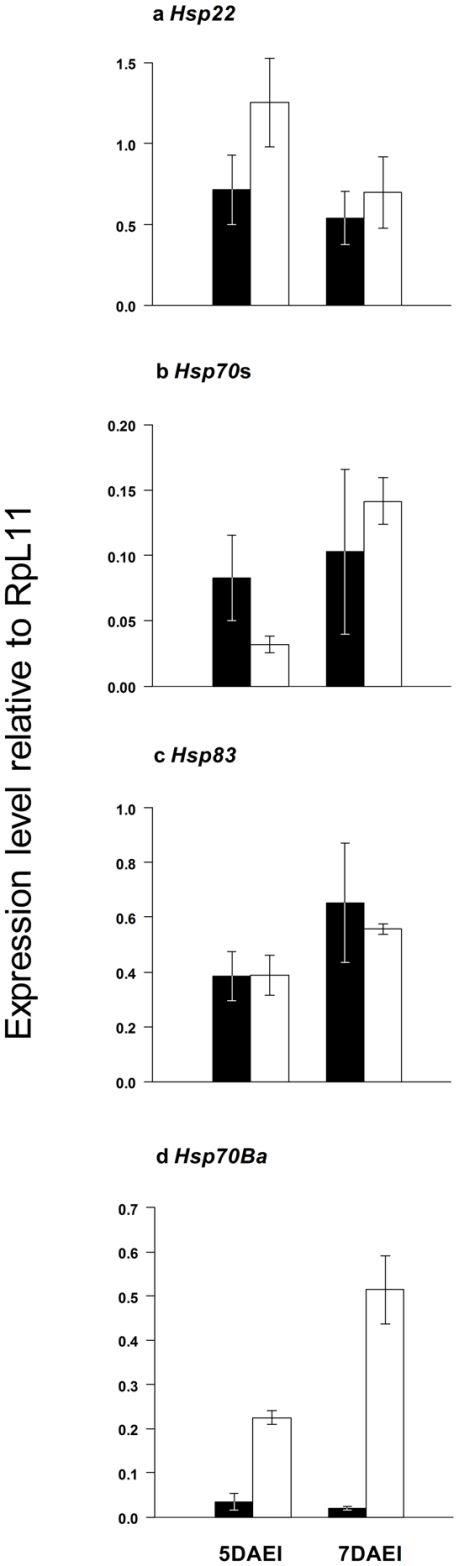
Level of *Hsp22*, *Hsp70*s, *Hsp83* and *Hsp70Ba* mRNA in larvae at 5 DAEI and 7 DAEI relative to RpL11 determined by real-time PCR. Closed bars are for control and open bars are for *Df(3R)ED5579*/DSK001. Error bars represent standard errors.

The expression levels of *Hsp22*, *Hsp70*s, and *Hsp83* were not significantly different between DSK001/DSK001 and *Df(3R)ED5579/*DSK001 at either 5 or 7 DAEI (Figure 2) under poor nutrition at 28°C. On the other hand, the expression level of *Hsp70Ba* was significantly higher in *Df(3R)ED5579/*DSK001 than in DSK001/DSK001 at 5 DAEI (*F* = 61.7; df = 1,4; *P* = 0.0014; Figure 2) and at 7 DAEI (*F* = 24.7; d.f. = 1, 3; *P* = 0.016; Figure 2).

### Repeatability and measurement error

Repeatability was quite high for the individual landmark coordinates (*R*>0.79) and very high for CS (*R*>0.99) (Table 2). Females tended to have higher repeatability than males and repeatability was similar in flies reared under constant conditions and those in the short-term heat stress experiments. The between-side variation of CS assessed as a two-way interaction term between side and individual was significantly larger than measurement error (*P*<0.0001) in all cases.

**Table 2:** Repeatability (*R*) in landmark coordinates and centroid size for constant condition and short-term heat stress experiments.

Experiments	Sex	Side	Average *R* for landmark coordinates	*R* for centroid size
Constant condition	Female	Right	0.846	0.999
		Left	0.856	0.999
	Male	Right	0.797	0.999
		Left	0.814	0.999
Short-term heat stress	Female	Right	0.896	0.999
		Left	0.900	0.999
	Male	Right	0.862	0.999
		Left	0.884	0.999

Procrustes ANOVA indicated that the contribution of measurement error to overall shape variation in general was small except for the case of females in short-term heat stress experiment (Table 3). Among-individual variation accounted for the largest portion of the total variation in most of the cases. The effect of FA was highly significant in all cases while that of DA was variable depending on sex and condition.

**Table 3:** Procrustes ANOVA for the wing landmarks.

			d.f.	SS	MS	*F*	*P*
Constant condition	Female	Individual	2220	84656.560	38.134	3.379	<0.0001
		Side	12	488.706	40.726	3.609	<0.0001
		Individual×Side	2220	25052.130	11.285	7.468	<0.0001
		Measurement error	4464	6745.890	1.511		
	Male	Individual	2136	63792.260	29.865	2.848	<0.0001
		Side	12	156.663	13.055	1.245	0.245782
		Individual×Side	2136	22401.070	10.487	6.055	<0.0001
		Measurement error	4296	7441.370	1.732		
Short-term heat stress	Female	Individual	2328	213120.520	91.547	1.931	<0.0001
		Side	12	720.630	60.053	1.267	0.231422
		Individual×Side	2328	110349.070	47.401	1.219	<0.0001
		Measurement error	4680	182008.010	38.891		
	Male	Individual	2352	123267.060	52.409	5.356	<0.0001
		Side	12	414.970	34.581	3.534	<0.0001
		Individual×Side	2352	23016.260	9.786	7.257	<0.0001
		Measurement error	4728	6375.420	1.348		

Sums of squares (SS) and mean squares (MS) are in dimensionless units of Procrustes distance. The sums of squares are added over landmarks and coordinates, assuming that all landmarks have the same amount of isotropic variation.

### Effect of treatments and deletions on trait means

**Figure 3:**
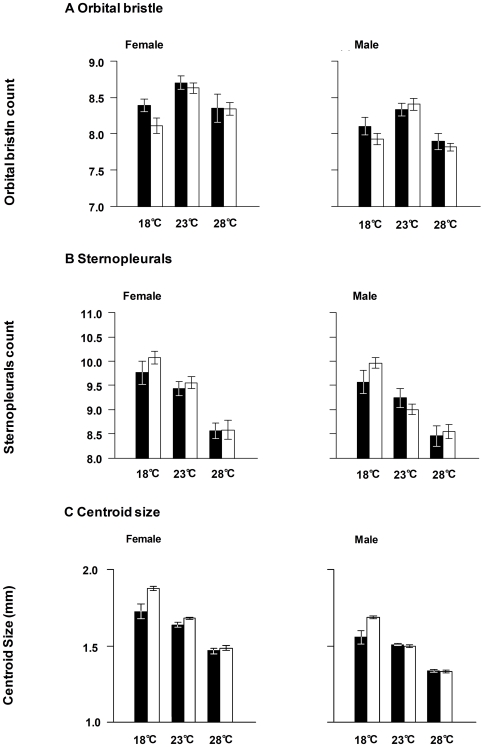
Effect of temperature and nutritional conditions on morphological traits. (A) mean numbers of orbital bristles, (B) numbers of sternopleural bristles, and (C) wing centroid size in mm for females and males in poor (closed bars) and rich (open bars) nutritional conditions under 18°C, 23°C, and 28°C. Error bars represent standard errors.

Poor nutrition and higher temperature generally reduced trait means (Figure 3). In the ANOVA on data from the constant condition experiment with *Df(3R)ED5579*/DSK001, temperature affected all traits in both sexes while nutrition had no significant effect. In the short-term heat stress experiment with the *Df(3R)ED5579*/DSK001, HST and the deletion did not have a significant effect on any trait. HST had no effect on any trait. An effect of side was not detected for any trait in either experiment.

The *Hsp70Ba^304^/Hsp70Ba^304^* genotype consistently reduced all the trait means in both constant and short-term heat stress conditions, and the effect was significant except for OR in the constant condition experiment (Table 4 and Table S1).

**Table 4:** Mean squares (from ANOVAs) for effects of environmental conditions and *Hsp70Ba* deletion in the constant condition experiment and the short-term heat stress experiment on mean orbital (OR) and sternopleural (SP) bristle numbers and wing size (CS).

			Female			Male		
Experiment		Source of variation	OR	SP	CS†	OR	SP	CS
*Df(3R)ED5579*/DSK001	Constant condition	Nut	0.19	0.07	3732.00	0.05	0.01	1364.00
		Temp	0.85*	17.25***	43876.00***	1.19***	7.03***	32962.00***
		Del	0.22	0.01	834.00	0.08	0.20	169.00
		Nut:Temp	0.11	0.22	1924.00	0.08	0.50	2684.00
		Nut:Del	0.10	0.00	519.00	0.25	0.01	593.00
		Temp:Del	0.16	0.28	426.00	0.21	0.22	737.00
		Nut:Temp:Del	0.09	2.17	1236.00	0.07	0.59	255.00
		Error	0.10	13.17	700.00	0.08	0.32	494.00
	Short-term heat stress	HST	0.14	0.15	85.12	0.00	0.16	41.13
		Del	0.18	0.27	502.38	0.11	0.01	270.79
		HST:Del	0.13	0.37	135.22	0.06	0.47	42.94
		Error	0.10	0.20	83.31	0.08	0.15	37.34
*Hsp70Ba^304^*/*Hsp70Ba^304^*	Constant condition‡	Del	1.31	4.84*	2238.76	1.57	4.68**	1567.75
		Error	0.07	0.10	46.29		0.04	38.93
	Short-term heat stress	HST	0.01	0.11	192.60	0.11	0.20	360.50*
		Del	4.92***	36.15***	20007.40***	4.41***	33.58***	19529.20***
		HST:Del	0.09	0.21	146.00	0.12	0.30	88.90
		Error	0.07	0.12	48.40	0.08	0.16	66.90

Nut: Nutrition (Poor, Rich), Temp: Temperature (18°C, 23°C, 28°C), Del: Deletion (Deletion, Control), HST: Heat Shock Timing (1, 3, 5, 7, and 9 days after egg introduction).

‡ Because of the very low viability under stressful conditions, only flies emerged at 23°C from the rich nutritional medium were measured.

† mulplied by 10^5^.

*P<0.05.

**P<0.01.

***P<0.001 after Bonferroni correction.

**Figure 4:**
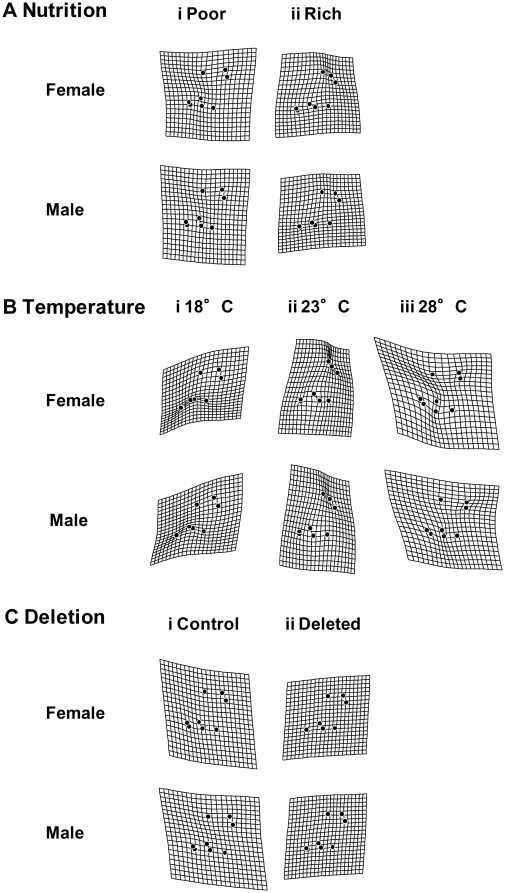
Thin-plate spline (x30) using eight landmarks between overall mean and mean configurations for wing shape of DSK001/DSK001 (Control) and *Df(3R)ED5579*/DSK001 (Deleted) genotypes in each treatment from the constant stress experiment.

**Figure 5:**
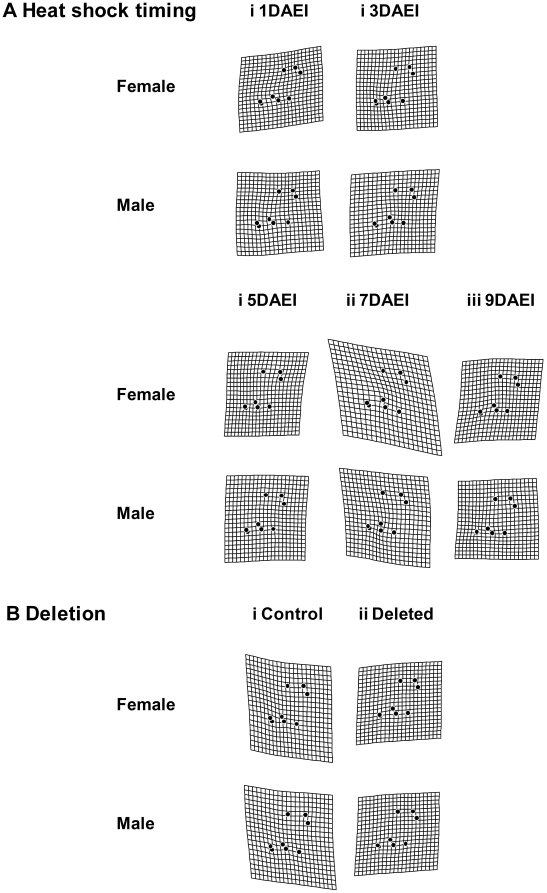
Thin-plate spline (x30) using eight landmarks between overall mean and mean configurations for wing shape of DSK001/DSK001 (Control) and *Df(3R)ED5579/*DSK001 (Deleted) in each treatment from the short-term heat stress experiment.

**Figure 6:**
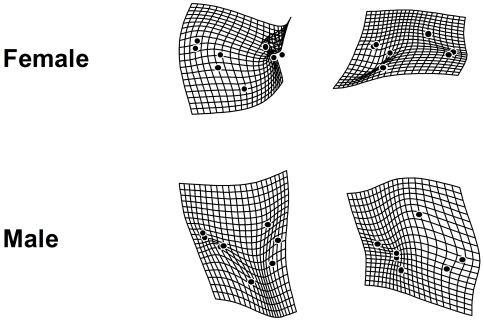
Thin-plate spline (x20) using eight landmarks between overall mean and mean configurations for wing shape of DSK001/DSK001 (Control) and *Hsp70Ba^304^/Hsp70Ba^304^* (Deleted) in the constant stress experiment.

**Figure 7:**
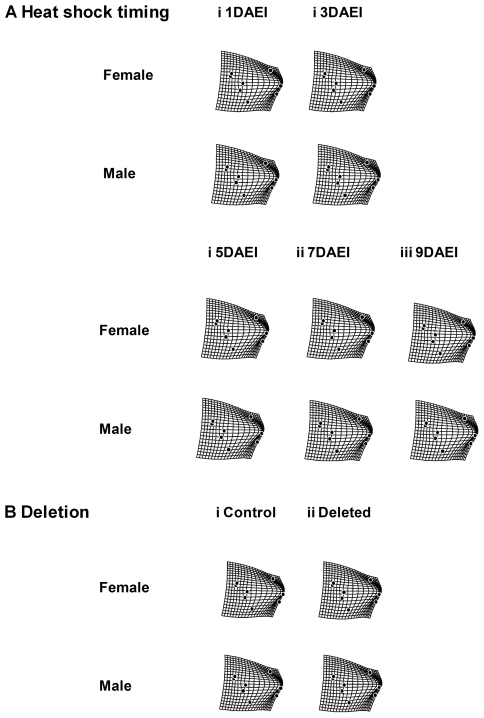
Thin-plate spline (x1) using eight landmarks between overall mean and mean configurations for wing shape of DSK001/DSK001 (Control) and *Hsp70Ba^304^*/*Hsp70Ba^304^* (Deleted) in each treatment from the short-term heat stress experiment.

The result of the thin-plate spline analysis for constant condition experiment with *Df(3R)ED5579*/DSK001 is shown in Figure 4. As a result of the comparison between overall mean wing shape and the mean wing shapes of each treatment, we could observe the largest deflection in “Temperature” compared to other treatments such as “Nutrition” and “Deletion”, indicating that “Temperature” had the largest contribution to wing shape variation as for the other morphological traits. The localization of the deflection (i.e., shape difference between overall mean and the mean of each treatment) differed depending on thermal conditions, while any sex difference in the localization of the deflection was very small (Figure 4). The thin-plate spline analysis for the short condition experiment with *Df(3R)ED5579*/DSK001 showed very weak deflection in any case, suggesting that neither HST nor deletion had a strong effect on mean wing shape (Figure 5). The thin-plate spline analysis for the constant condition experiment with *Hsp70Ba^304^*/*Hsp70Ba^304^* showed very strong deflection in all cases (Figure 6). This indicates that the shape difference between overall mean wing shape and the mean wing shape of each genotype was large in both sexes, suggesting substantial between-genotype variation in wing shape. In the case of the short exposure experiment with *Hsp70Ba^304^*/*Hsp70Ba^304^*, the degree of deflection was substantial in all cases (Figure 7). In this graphical display, we did not magnify the shape difference between the overall mean and those of each treatment given the substantial effects observed.

### Effect of treatments and deletion on CV

In the constant stress experiment, *Df(3R)ED5579*/DSK001 did not have a significant effect on CVs of any traits, while temperature and the three-way interaction among temperature, nutrition, and deletion had a significant effect. The *Hsp70Ba^304^/Hsp70Ba^304^* genotype had significantly higher CV of OR in females (mean for *Hsp70Ba^304^/Hsp70Ba^304^* and control, 0.110+/−0.014 and 0.072+/−0.011 respectively) (Table 5 and Table S2). In the short-term heat stress experiment, *Df(3R)ED5579*/+ did not have a significant effect on CVs of any traits (Table 5). On the other hand, *Hsp70Ba^304^/Hsp70Ba^304^* had a significantly higher CV of SP in females (mean for *Hsp70Ba^304^/Hsp70Ba^304^* and control, 0.116+/−0.02 and 0.095+/−0.03), and also significantly higher CS in females (mean for *Hsp70Ba^304^/Hsp70Ba^304^* and control, 0.030+/−0.004 and 0.022+/−0.005 respectively) (Table 5 and Table S2). HST also had a significant effect in the experiment with *Hsp70Ba^304^/Hsp70Ba^304^* (Table 4).

**Table 5:** Mean squares (from ANOVAs) multipled by 10^5^ for effects of environmental conditions and *Hsp70Ba* deletion in the constant condition experiment and the short-term heat stress experiment on CV of orbital (OR) and sternopleural (SP) bristle numbers and wing size (CS).

			Female			Male		
Experiment		Source of variation	OR	SP	CS	OR	SP	CS
*Df(3R)ED5579*/DSK001	Constant condition	Nut	104.11	238.69	72.07	5.88	451.90	58.19
		Temp	24.29	131.94	140.58	22.34	240.10	196.88**
		Del	0.45	197.42	97.68	94.58	7.10	37.14
		Nut:Temp	114.07	23.86	142.65	14.22	167.40	101.89
		Nut:Del	93.96	0.97	24.47	0.03	16.20	98.39
		Temp:Del	102.39	147.32	4.11	94.11	135.00	31.06
		Nut:Temp:Del	61.51	78.00	41.37	121.82	119.50	389.98***
		Error	47.04	50.05	21.05	34.03	76.30	20.92
	Short-term heat stress	HST	94.78	50.00	12.84†	35.81	41.22	5.19†
		Del	72.89	42.84	27.07†	38.28	65.54	5.65†
		HST:Del	34.13	101.97	10.64†	88.84	127.03	5.38†
		Error	29.20	52.09	12.64†	35.64	37.49	6.59†
*Hsp70Ba^304^*/*Hsp70Ba^304^*	Constant condition‡	Del	356.89*	215.02	0.86	208.35	203.77	0.81
		Error	16.78	72.07	2.66	27.84	33.89	10.58
	Short-term heat stress	HST	110.44	262.76**	2.10	140.59	254.77	5.01
		Del	246.86	477.71**	67.07***	189.62	381.64	65.45
		HST:Del	55.43	53.67	3.12	10.89	114.87	16.57
		Error	40.02	35.20	1.93	47.77	63.02	6.93

Nut: Nutrition (Poor, Rich), Temp: Temperature (18°C, 23°C, 28°C), Del: Deletion (Deletion, Control), HST: Heat Shock Timing (1, 3, 5, 7, and 9 days after egg introduction).

‡ Because of the very low viability under stressful conditions, only flies emerged at 23°C from the rich nutritional medium were measured.

† not mulplied by 10^5^.

*P<0.05.

**P<0.01.

***P<0.001 after Bonferroni correction.

### Effect of treatments and deletion on FA

In both the constant condition experiment and the short-term stress experiment, we detected no significant effect of *Df(3R)ED5579*/DSK001 or *Hsp70Ba^304^/Hsp70Ba^304^* on FA of any trait after Bonferroni correction of probabilities (Table 6 and Table S3).

**Table 6:** Mean squares (from ANOVAs) multipled by 10^5^ for effects of environmental conditions and *Hsp70Ba* deletion in the constant condition experiment and the short-term heat stress experiment on FA of orbital (OR) and sternopleural (SP) bristle numbers and wing size (CS), and wing shapes (WS1 and WS2).

			Female					Male				
Experiment		Source of variation	OR	SP	CS	WS1†	WS2†	OR	SP	CS	WS1†	WS2†
*Df(3R)ED5579*/DSK001	Constant condition	Nut	210.30	282.80	0.49	0.02	1.53	35.20	154.10	0.61	0.41	5.780
		Temp	0.20	1304.10	0.02	0.01	7.41	841.20	1002.10	1.01	0.02	2.640
		Del	0.80	104.60	0.01	0.57	49.98	328.60	596.40	0.18	0.02	3.310
		Nut:Temp	67.70	336.90	0.08	0.95	10.33	10.50	918.20	0.09	0.10	14.140
		Nut:Del	73.90	0.20	0.07	0.74	10.40	36.10	1196.10	2.58	0.33	2.850
		Temp:Del	560.40	140.80	0.08	0.17	0.03	77.30	719.70	0.07	0.10	20.500
		Nut:Temp:Del	41.40	211.50	0.04	0.45	12.29	313.70	883.10	0.42	0.72	4.670
		Error	151.10	271.60	0.27	0.48	7.84	194.00	477.80	0.59	0.19	8.440
	Short term heat stress	HST	284.70	604.00	0.75	0.09	0.10	113.90	404.20	0.39	0.00	0.574
		Del	9.50	380.70	0.08	0.14	6.63	3.60	1.70	0.37	0.01	0.505
		HST:Del	121.20	337.40	0.27	0.07	1.72	201.80	617.90	0.47	0.01	5.013
		Error	308.40	193.70	1.11	0.21	7.03	197.70	275.70	0.62	0.16	4.141
*Hsp70Ba^304^*/*Hsp70Ba^304^*	Constant condition‡	Del	290.66	116.08	0.37	0.00	40.86	46.16	590.80	0.15	0.07	6.89
		Error	43.57	423.02	0.02	0.03	12.92	157.35	244.57	0.02	0.03	27.06
	Short term heat stress	HST	80.40	336.70	0.03	0.32	60.96	381.50	304.60	0.01	0.39	50.84
		Del	208.40	233.20	0.18	0.03	29.49	288.80	215.40	0.51	0.28	30.33
		HST:Del	301.10	438.00	0.16	0.25	37.76	99.50	475.90	0.04	0.52	18.89
		Error	333.40	233.10	0.08	0.21	19.75	322.10	470.20	0.06	0.22	37.32

Nut: Nutrition (Poor, Rich), Temp: Temperature (18°C, 23°C, 28°C), Del: Deletion (Deletion, Control), HST: Heat Shock Timing (1, 3, 5, 7, and 9 days after egg introduction).

† not mulplied by 10^5^.

‡ Because of the very low viability under stressful conditions, only flies emerged at 23°C from the rich nutritional medium were measured.

*P<0.05.

**P<0.01.

***P<0.001 after Bonferroni correction.

## Discussion

The significant effect of the *Hsp70Ba* deletion heterozygote and homozygote on means of morphological traits, and the effect of the deletion homozygote on CV of some morphological traits, may be related to changes in the expression level of *Hsp70Ba*. The expression of *Hsp70Ba* was six to 20 times higher in *Df(3R)ED5579/*DSK001, while the expression levels of other *Hsp* genes, *Hsp22*, *Hsp70*s, and *Hsp83,* were not different between the control homozygotes and *Df(3R)ED5579/*DSK001. The induction of *Hsp* transcripts is mediated by the heat shock transcription factor (HSF) [52]. The mechanism of activation of HSF has been intensively studied, and various factors such as pH and temperature are known to affect the activation of HSF [53], [54]. In the present study, the mechanism of over-expression of *Hsp70Ba* in the *Df(3R)ED5579/*DSK001 is unclear, but it is possible that the deletion activated HSF. Somatic pairing of homologous chromosomes may explain the hyperactivation of *Hsp70Ba*. The pairing of homologous chromosomes occurs in mitotic cells of *Drosophila*, and transvection is an example of pairing-dependent inter-allelic interactions [55]. Failure of chromosome pairing might lead to improper regulation of *Hsp70Ba* gene expression and hence hyperactivation in the *Df(3R)ED5579/*DSK001.

However, it is also possible that genetic background effects were partly responsible for the results. In the *Df(3R)ED5579/*DSK001, several other genes were missing, including one gene thought to be involved in stress responses. In the deletion homozygote, we controlled for differences in the X and 2nd chromosomes but not the 3rd chromosome. The very low viability of the deletion homozygote under thermal and nutritional stress suggests an effect of *Hsp70Ba* on stress resistance. Additional data are required to assign these effects specifically to *Hsp70Ba*.

Williams *et al.* [31] observed an increased rate of developmental abnormalities of discrete traits in strains with extra-copies of *Hsp70*. A harmful effect of excessive expression of *Hsp70* on fitness was also observed by Krebs and Feder [56], [57]. Williams *et al.* [31] suggested that while induction of HSP70 may help keep hyperthermic cells alive, it may also overstimulate or inhibit numerous signaling pathways involved in cell proliferation, maturation and death, resulting in developmental failure [58]. In the current study, only the absence of *Hsp70Ba* expression was associated with changes in means; CVs were only modified by an absence of expression in 3 out of 12 tests and FA was not altered. We did not observe morphological wing abnormalities. Therefore quite large changes in *Hsp70Ba* expression have occurred with only isolated effects on canalization. Part of the reason for the limited effect of the *Hsp70Ba* deletion might be because of a high level of basal FA in the control strain due to homozygosity. Although Fowler and Whitlock [59] reported that moderate inbreeding did not affect FA in *D. melanogaster*, severe inbreeding could affect FA and obscure deletion effects. A potential explanation for the lack of an effect of elevated *Hsp70Ba* expression level on morphological traits is that the expression level of mRNA did not directly reflect HSP70Ba protein level. The complicated regulatory mechanisms of *Hsp70* function in the cell might buffer phenotypic effects of high *Hsp70Ba* expression levels.

Effects of nutritional and thermal stresses on bristle numbers and wing variability including FA have been observed in many studies [60], [61], [62], [63], [64]. In the present study, temperature had a large effect on mean trait values in general, while nutrition influenced wing morphology. There was an effect of nutrition on the pre-adult period and viability to adult stage for both males and females, which may have influenced the distribution of morphological traits through selective mortality. However this was not the case for temperature. An effect of side was detected only in wing landmarks under limited conditions, suggesting that fluctuating rather than directional asymmetry is important.

The primary functions of HSP90 and HSP70 are to buffer the phenotype against environmental perturbations. If this function is beneficial, a general buffering mechanism that covers a wide range of environmental perturbations would be favored by natural selection. However, *Hsp90* and *Hsp70* are limited in their ability to buffer environmental perturbations. Bergman and Siegal [65] and Milton *et al.* [9] suggested that global regulation of canalization and developmental stability by *Hsp90* is unlikely, and there could be other independent and idiosyncratic buffering mechanisms. In fact, Takahashi et al. [66] found that four small *Hsp* genes (*Hsp22*, *Hsp67Ba, Hsp67Bb* and *Hsp67Bc*) were involved in the process of morphogenesis and developmental stability in a trait-specific manner. The current study suggests that *Hsp70Ba* could be associated with a developmental buffering mechanism with a different function to *Hsp90* given the detected effects on CVs of some traits.

In the short-term heat stress experiment, heat shock timing had a significant effect on a wing trait as in the previous study [31], but the effect was only detected in the male deletion homozygote. This result suggests that *Hsp70Ba* may influence a wing trait at specific times during the course of development.

In the current study, the absence of the *Hsp70Ba* in deletion homozygote impaired canalization defined as among-individual variation, but not developmental stability defined as within-individual variation. The link between canalization and developmental stability has been controversial. Some researchers suggested that they are regulated by at least partly different mechanisms [9], [67], [68], [69]. The current results support this view, and suggest that *Hsp70Ba* may have a trait-specific effect on canalization but not on developmental stability. The new findings suggest but do not yet conclusively demonstrate that molecular chaperones other than HSP90 influence canalization. Further studies are needed to isolate the effects of particular molecular chaperones in the canalization processes.

## Supporting Information

Table S1 **Mean scores of orbital (OR) and sternopleural (SP) bristle numbers and wing size (CS) under different nutritional and thermal conditions in this study.** Standard errors of the estimation of the means are in the in parentheses.
(PDF)

Table S2 **Mean CV values for orbital (OR) and sternopleural (SP) bristle numbers and wing size (CS) under different nutritional and thermal conditions in this study.** Standard errors of the estimation of the means are in the in parentheses.
(PDF)

Table S3 **Mean FA of orbital (OR) and sternopleural (SP) bristle numbers and wing size (CS) two wing shapes (WS1 and WS2) under different nutritional and thermal conditions in this study.** Standard errors of the estimation of the means are in the in parentheses.
(PDF)
