# Supplementary material for: Environmental Stress-Dependent Effects of Deletions Encompassing Hsp70Ba on Canalization and Quantitative Trait Asymmetry in Drosophila melanogaster
Source: PLoS One. 2011 Apr 25;6(4):e17295. doi: 10.1371/journal.pone.0017295 (PMC3081816; doi:10.1371/journal.pone.0017295)
Supplement: Table S3 — Mean FA of orbital (OR) and sternopleural (SP) bristle numbers and wing size (CS) two wing shapes (WS1 and WS2) under different nutritional and thermal conditions in this study. Standard errors of the estimation of the means are in the in parentheses. (PDF) [file pone.0017295.s003.pdf]

Table S3. Mean FA of orbital (OR) and sternopleural (SP) bristle numbers and wing size (CS) two wing shapes (WS1 and WS2) under different nutritional and thermal conditions in this study. Standard errors of the estimation of the means are in the in parentheses.

Genotype	Experiment	sex	Nutirition	Temperature	OR	SP	CS	WS1	WS2
DSK001/DSK001	Constant condition	Female	Poor	18°C	0.112 (0.019)	0.056 (0.023)	0.006 (0.001)	3.187 (0.279)	0.041 (0.004)
				23°C	0.051 (0.014)	0.098 (0.033)	0.006 (0.001)	2.625 (0.223)	0.038 (0.005)
				28°C	0.067 (0.014)	0.123 (0.003)	0.007 (0.002)	3.419 (0.566)	0.042 (0.005)
			Rich	18°C	0.094 (0.013)	0.056 (0.031)	0.005 (0.001)	3.292 (0.313)	0.046 (0.002)
				23°C	0.052 (0.013)	0.148 (0.037)	0.004 (0.001)	3.196 (0.321)	0.043 (0.002)
				28°C	0.090 (0.035)	0.102 (0.028)	0.005 (0.001)	3.623 (0.158)	0.047 (0.005)
		Male	Poor	18°C	0.109 (0.015)	0.137 (0.042)	0.004 (0.001)	3.055 (0.319)	0.048 (0.006)
				23°C	0.080 (0.024)	0.112 (0.058)	0.005 (0.000)	3.035 (0.275)	0.041 (0.003)
				28°C	0.087 (0.043)	0.179 (0.029)	0.006 (0.001)	3.127 (0.169)	0.047 (0.007)
			Rich	18°C	0.121 (0.024)	0.139 (0.018)	0.008 (0.001)	3.915 (0.347)	0.056 (0.003)
				23°C	0.038 (0.021)	0.137 (0.023)	0.006 (0.002)	3.020 (0.111)	0.042 (0.004)
				28°C	0.126 (0.002)	0.239 (0.028)	0.010 (0.002)	3.515 (0.196)	0.052 (0.004)
	Short-term stress	Female	Rich	1DAEI	0.121 (0.061)	0.106 (0.022)	0.005 (0.002)	3.302 (0.156)	0.046 (0.005)
				3DAEI	0.054 (0.012)	0.153 (0.041)	0.007 (0.001)	3.499 (0.315)	0.046 (0.006)
				5DAEI	0.079 (0.016)	0.056 (0.011)	0.005 (0.001)	3.208 (0.191)	0.045 (0.006)
				7DAEI	0.054 (0.036)	0.097 (0.034)	0.007 (0.001)	3.499 (0.312)	0.049 (0.002)
				9DAEI	0.093 (0.015)	0.162 (0.023)	0.007 (0.003)	3.259 (0.382)	0.043 (0.004)
		Male	Rich	1DAEI	0.064 (0.046)	0.147 (0.012)	0.006 (0.001)	3.227 (0.450)	0.043 (0.006)
				3DAEI	0.099 (0.025)	0.081 (0.023)	0.003 (0.002)	3.224 (0.220)	0.039 (0.003)
				5DAEI	0.059 (0.034)	0.097 (0.028)	0.008 (0.003)	3.536 (0.241)	0.049 (0.005)
				7DAEI	0.073 (0.021)	0.122 (0.024)	0.004 (0.000)	3.157 (0.330)	0.039 (0.001)
				9DAEI	0.077 (0.044)	0.160 (0.012)	0.004 (0.001)	3.275 (0.068)	0.040 (0.004)
Df(3R)ED5579 /DSK001	Constant condition	Female	Poor	18°C	0.058 (0.016)	0.062 (0.011)	0.008 (0.002)	3.897 (0.545)	0.055 (0.005)
				23°C	0.089 (0.019)	0.111 (0.039)	0.005 (0.001)	3.268 (0.232)	0.048 (0.003)
				28°C	0.062 (0.019)	0.138 (0.015)	0.005 (0.001)	3.386 (0.182)	0.049 (0.003)
			Rich	18°C	0.077 (0.017)	0.106 (0.016)	0.007 (0.001)	3.091 (0.125)	0.049 (0.003)
				23°C	0.090 (0.013)	0.114 (0.012)	0.006 (0.002)	3.115 (0.174)	0.041 (0.003)
				28°C	0.090 (0.010)	0.116 (0.027)	0.009 (0.001)	3.691 (0.170)	0.054 (0.002)
		Male	Poor	18°C	0.091 (0.021)	0.135 (0.017)	0.007 (0.001)	3.286 (0.131)	0.048 (0.004)
				23°C	0.053 (0.013)	0.122 (0.020)	0.006 (0.001)	3.636 (0.260)	0.054 (0.006)
				28°C	0.100 (0.015)	0.198 (0.028)	0.006 (0.001)	3.003 (0.186)	0.046 (0.004)
			Rich	18°C	0.079 (0.020)	0.086 (0.013)	0.005 (0.001)	3.113 (0.089)	0.048 (0.003)
				23°C	0.061 (0.014)	0.167 (0.059)	0.006 (0.001)	3.185 (0.184)	0.048 (0.003)
				28°C	0.078 (0.010)	0.089 (0.010)	0.004 (0.001)	3.685 (0.094)	0.053 (0.005)
	Short-term stress	Female	Rich	1DAEI	0.099 (0.016)	0.141 (0.024)	0.007 (0.001)	3.423 (0.353)	0.048 (0.006)
				3DAEI	0.081 (0.022)	0.066 (0.018)	0.005 (0.001)	3.304 (0.263)	0.045 (0.002)
				5DAEI	0.050 (0.015)	0.126 (0.023)	0.009 (0.002)	3.384 (0.142)	0.048 (0.002)
				7DAEI	0.058 (0.026)	0.088 (0.015)	0.006 (0.001)	3.247 (0.272)	0.043 (0.003)
				9DAEI	0.101 (0.024)	0.081 (0.011)	0.007 (0.001)	3.817 (0.176)	0.048 (0.003)
				1DAEI	0.092 (0.027)	0.128 (0.020)	0.005 (0.001)	3.507 (0.154)	0.045 (0.002)
		Male	Rich	3DAEI	0.090 (0.014)	0.072 (0.011)	0.004 (0.001)	3.116 (0.245)	0.043 (0.003)
				5DAEI	0.075 (0.006)	0.109 (0.021)	0.005 (0.001)	3.417 (0.158)	0.041 (0.002)
				7DAEI	0.088 (0.008)	0.129 (0.027)	0.006 (0.001)	3.411 (0.193)	0.046 (0.001)
				9DAEI	0.066 (0.012)	0.131 (0.029)	0.005 (0.001)	3.461 (0.174)	0.046 (0.002)
DSK001/DSK001 for the comparison with *Hsp70Ba*^{304}	Constant condition	Female	Rich	23°C	-	-	-	3.431 (0.074)	24.217 (1.136)
		Male	Rich	23°C	-	-	-	3.453 (0.102)	21.841 (2.849)
	Short-term stress	Female	Rich	1DAEI	-	-	-	3.262 (0.505)	24.758 (3.645)
				3DAEI	-	-	-	3.197 (0.211)	22.702 (2.061)
				5DAEI	-	-	-	3.485 (0.206)	25.297 (3.509)
				7DAEI	-	-	-	3.205 (0.373)	22.980 (2.498)
				9DAEI	-	-	-	3.348 (0.040)	23.146 (3.178)
		Male	Rich	1DAEI	-	-	-	3.345 (0.188)	27.020 (2.643)
				3DAEI	-	-	-	3.457 (0.322)	25.216 (3.357)
				5DAEI	-	-	-	3.188 (0.206)	22.783 (2.815)
				7DAEI	-	-	-	3.352 (0.226)	24.974 (1.426)
				9DAEI	-	-	-	3.091 (0.332)	23.522 (2.668)
Hsp70Ba^{304}/*Hsp70Ba*^{304}	Constant condition	Female	Rich	23°C	0.093 (0.010)	0.122 (0.033)	0.002 (0.001)	3.446 (0.098)	29.100 (2.176)
		Male	Rich	23°C	0.054 (0.021)	0.078 (0.027)	0.002 (0.000)	3.255 (0.079)	23.845 (2.686)
	Short-term stress	Female	Rich	1DAEI	0.084 (0.017)	0.102 (0.027)	0.002 (0.001)	3.061 (0.234)	24.479 (2.005)
				3DAEI	0.067 (0.015)	0.114 (0.013)	0.002 (0.000)	3.024 (0.158)	23.079 (2.127)
				5DAEI	0.136 (0.038)	0.073 (0.022)	0.001 (0.000)	3.323 (0.140)	23.764 (0.927)
				7DAEI	0.073 (0.027)	0.153 (0.038)	0.002 (0.001)	3.886 (0.245)	32.339 (1.264)
				9DAEI	0.084 (0.030)	0.075 (0.027)	0.002 (0.000)	3.549 (0.151)	28.792 (1.594)
		Male	Rich	1DAEI	0.099 (0.045)	0.141 (0.044)	0.003 (0.000)	3.512 (0.316)	30.737 (5.592)
				3DAEI	0.079 (0.022)	0.107 (0.024)	0.002 (0.001)	3.178 (0.255)	25.184 (2.851)
				5DAEI	0.100 (0.023)	0.139 (0.035)	0.002 (0.000)	4.240 (0.136)	29.994 (2.000)
				7DAEI	0.075 (0.022)	0.107 (0.011)	0.002 (0.000)	3.200 (0.185)	25.294 (2.034)
				9DAEI	0.137 (0.024)	0.139 (0.065)	0.002 (0.000)	3.162 (0.276)	23.663 (3.422)
